# Analysis of characteristics among unemployed callers to the psychological support hotline in Beijing

**DOI:** 10.3389/fpsyt.2025.1658354

**Published:** 2025-08-25

**Authors:** Xingxue Li, Zikang Liu, Tong Wu, Zhe Yuan, Wei Wang, Liting Zhao, Shuchang Yang, Hong Liang

**Affiliations:** ^1^ Beijing Suicide Research and Prevention Center, Beijing Huilongguan Hospital, Beijing, China; ^2^ World Health Organization Collaborating Center for Research and Training in Suicide Prevention, Beijing, China; ^3^ Graduate School, Chengde Medical University, Chengde, China; ^4^ Faculty of Psychology, Tianjin Normal University, Tianjin, China; ^5^ Mental Health Education Center, Baoding Institute of Technology, Baoding, Hebei, China; ^6^ Psychology and Language Science, Univeristy College London, London, United Kingdom

**Keywords:** psychological support hotline, unemployment, mental health, characteristics, risk factors

## Abstract

**Introduction:**

Unemployment constitutes a significant social stressor that systematically impairs individuals’ psychological adaptive capacity. It serves as an independent risk factor for mental health deterioration, significantly elevating the risks of depressive disorders, suicidal ideation, and related conditions. This study analyzes characteristics and mental health status of unemployed callers through psychological support hotline data, aiming to identify underlying psychological risk factors.

**Methods:**

A retrospective analysis was conducted on the general demographic data, primary concerns, and mental health status of unemployed callers to the Beijing Psychological Support Hotline from 2020 to 2023. Group comparisons were stratified by age and gender. Logistic regression models were used to identify factors associated with mental health outcomes.

**Results:**

The study included 9,002 call records from unemployed individuals, with 60.3% being female, 70.1% aged between 19 and 29, 73.2% unmarried, and 64.8% having no more than 15 years of education. Callers aged 19–29 years reported significantly higher proportions of non-familial interpersonal issues (*χ*²=8.03*, P*<0.01), depressive symptoms (*χ*²=7.48*, P*<0.01), and psychiatric symptoms (*χ*²=7.04*, P*<0.01) compared to those aged 30–59 years. Female callers (OR=1.50) exhibited higher susceptibility to suicidal ideation, hopelessness, and depression; individuals aged 30–59 years (OR=0.34-0.80*, P*<0.05) and those with prior psychiatric diagnoses (OR=0.66) demonstrated lower suicidal ideation risk; unmarried/divorced callers showed elevated hopelessness (OR=1.30-1.63), psychological distress (OR=1.39) and depression (OR=1.57); callers with ≥it years of education had lower levels of hopelessness, distress, and depression (OR=0.68-0.89*, P*<0.05); and daytime callers (8:00-23:00) exhibited lower hopelessness levels (OR=0.68-0.80*, P*<0.05).

**Conclusions:**

Unemployed callers who are female, aged 19–29 or unmarried/divorced exhibited higher levels of psychological distress and suicide risk. Notably, while callers with a history of psychiatric diagnoses reported lower levels of suicidal ideation, they demonstrated higher levels of hopelessness and depressive symptoms. These findings warrant attention, and hotline services should implement timely identification, psychological support, and targeted interventions to effectively alleviate psychological distress and reduce potential suicide risk in these high-risk groups.

## 1 Introduction

Employment, as a core element of socioeconomic development, is not only a critical pathway for individuals to achieve economic independence and self-fulfillment but also a cornerstone for national stability and prosperity. Employment provides individuals with a stable income source, facilitates career development, maximizes personal potential (1), and enhances self-identity and sense of social belonging (2). From a societal perspective, full employment contributes to expanding domestic demand, reducing social inequality, and maintaining social harmony and stability (3).

In recent years, rapid changes in the global economic environment and labor market instability have made unemployment a critical issue in the field of global public health. Multiple shocks, such as the COVID-19 pandemic, geopolitical conflicts, and technological transformations, have resulted in uneven global economic recovery and exacerbated unemployment issues. The ILO’s *World Employment and Social Outlook: Trends 2025* report indicates that global economic growth is slowing, making a full recovery of labor markets increasingly difficult. In 2024, global employment grew in line with labor force expansion, with the unemployment rate stabilizing at 5%. However, youth unemployment showed minimal improvement, remaining at 12.6% (4). Against the backdrop of economic restructuring and intensifying market competition, many individuals are confronting job instability and employment challenges.

Unemployment has profound negative effects on individuals’ mental health (5).Studies showed shown that economic stressors such as unemployment are major contributors to depression, anxiety, substance abuse, suicide, and other mental health disorders (6). The prevalence of depression, anxiety, and suicidal ideation is significantly higher among the unemployed compared to those who are employed, with mental health declining as the duration of unemployment increases (7). Long-term unemployed individuals are particularly prone to complex psychological issues, such as diminished self-worth, increased feelings of social isolation, and heightened uncertainty about the future, which may further develop into a potential psychological crisis (8).Moreover, the decline in economic circumstances adversely affects not only unemployed individuals but also their family members within the same household (9).

Jahoda’s latent deprivation model offers a robust theoretical framework for understanding the psychological distress experienced by unemployed populations (10, 11). Jahoda posits that unemployment impairs mental health not only through the loss of its manifest economic function but, more critically, through the deprivation of five latent psychological functions: temporal structure, social interaction, collective purpose, social status, and goal-directed activity (12). In the absence of employment, individuals may fall into an ‘existential vacuum’, which is characterized by temporal disorientation, social withdrawal, impaired self-esteem, and a reduced sense of purpose. Longitudinal research highlights a cumulative effect: initial stages of unemployment are often associated with anxiety and depression, whereas chronic unemployment can result in emotional blunting and impaired social functioning (13).

Furthermore, Durkheim’s theory of suicide further explained the association between unemployment and suicidal ideation, Positing that weakened social integration during unemployment fosters anomie, thereby increasing the risk of suicide (14). Thus, integrating Jahoda’s latent deprivation model with Durkheim’s sociological framework offers a comprehensive lens for interpreting the multifactorial effects of unemployment on mental health.

The Beijing Psychological Support Hotline was launched at the end of 2002 and has since become one of the largest services of its kind (15), receiving over 600,000 calls nationwide. As a public welfare psychological support platform, the hotline offers extensive call coverage, professionally trained operators, and highly confidential and immediate intervention channels (16). This enables callers to freely express their inner emotions and distress (17), while also providing a unique real-time data source for studying the hotline caller population. This study focuses on unemployed callers to the psychological support hotline, aiming to investigate their characteristics, primary concerns, mental health status, and influencing factors, thereby providing mental health professionals with evidence-based insights to better understand this population and inform targeted intervention strategies.

## 2 Materials and methods

### 2.1 Sample

**Figure 1:**
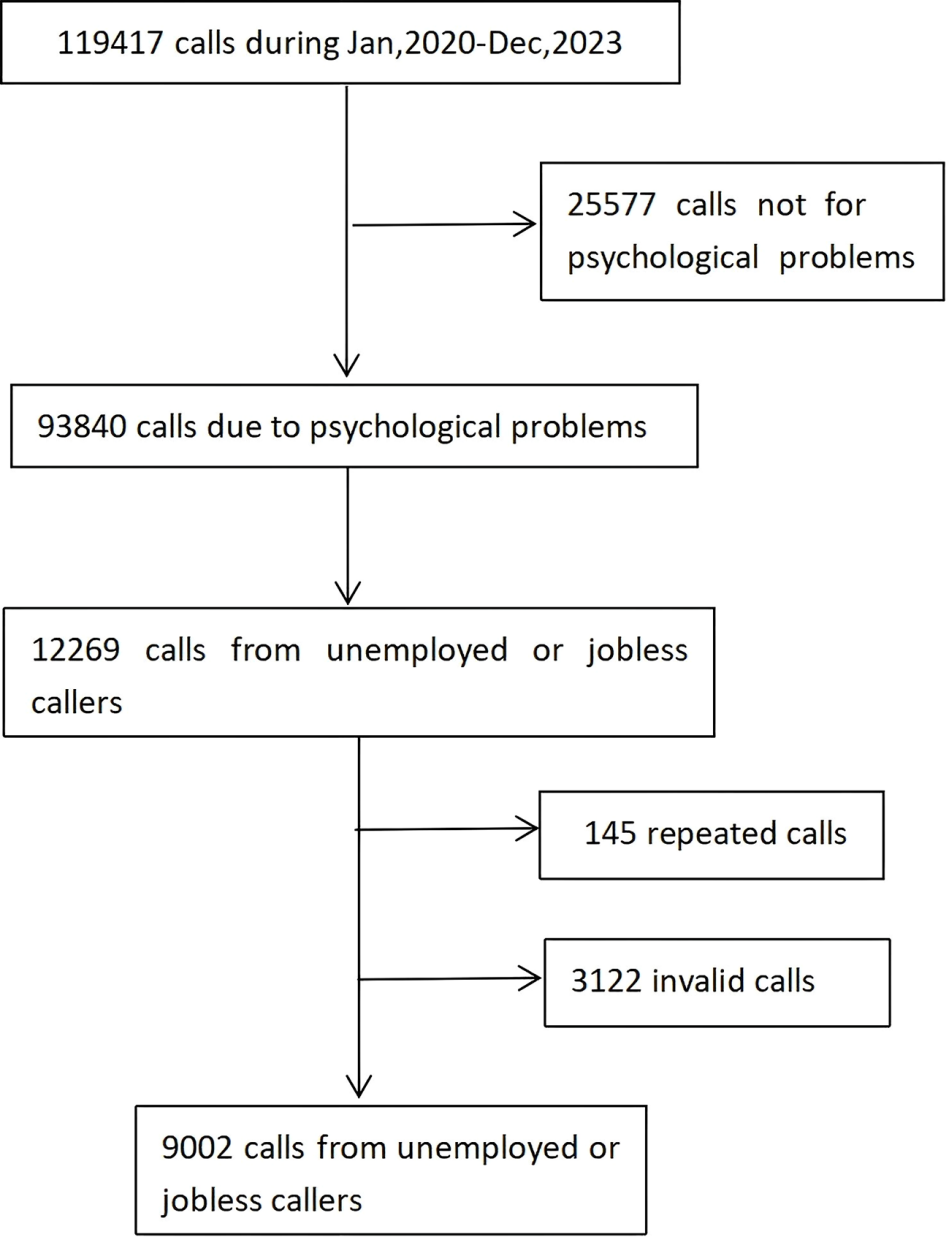
Flowchart of enrolling and screening callers.

The process for selecting unemployed callers is shown in **Figure 1**. This current study initially included a total of 12,269 callers to the Beijing Psychological Support Hotline from January 2020 to December 2023 who were identified as unemployed or not currently working. After excluding 3,122 invalid calls (e.g., silence, harassment, or calls lasting less than 10 minutes as such brief interactions typically lack sufficient information for reliable assessment of mental health status) and 145 duplicate calls (retaining only one record per caller based on completeness and earliest timestamp), the final sample consisted of 9,002 calls.

The Ethics Committee of Beijing Huilongguan Hospital approved this study (Approval No. 2022-12-Science). All callers were informed at the beginning of the call that the conversation would be recorded and that the data would be used solely for research and educational purposes; calls proceeded only upon obtaining verbal consent.

The Beijing Psychological Support Hotline employs a comprehensive evaluation system in which operators create service records, collect demographic data through interviews, and assess and record suicide-related risk factors (18). Furthermore, based on the callers’ primary concerns during consultations, the main topics were categorized into the following domains: family relationship issues, interpersonal issues outside the family, employment-related problems, financial problems, academic-related issues, depressive symptoms, psychiatric symptoms, and other adverse life events.

### 2.2 Measures

Hotline operators evaluated whether a caller identified as a survivor of suicide loss had experienced suicidal ideation in the preceding two weeks by asking, “Have you had any thoughts of suicide during the past two weeks?” If the caller reported no suicidal ideation over the past two weeks, they were classified into the no suicidal ideation group. If the caller reported having experienced suicidal ideation in the past two weeks, they were classified into the suicidal ideation group (16).

Hotline operators assessed callers’ depressive symptoms using the Depression Diagnostic Screening Scale (DSID) (19), which was developed based on the nine criteria for major depressive episodes in the Diagnostic and Statistical Manual of Mental Disorders, Fourth Edition (DSM-IV) (20). The DSID has been validated in Chinese populations, demonstrating 96.8% diagnostic accuracy and a Kappa coefficient of 0.87 against the Structured Clinical Interview for DSM Disorders (SCID) (19). In this study, the DSID was used to evaluate the severity and duration of depressive symptoms during the preceding month. The total score ranges from 0 to 100, with higher scores indicating more severe depressive symptoms. In this study, depressive mood scores were dichotomized at the median into a high depressive mood group (50–100) and a low depressive mood group (0–49).

The distress level was assessed via self-report, where callers were asked to evaluate the intensity of distress they experienced in relation to the current event. The score ranges from 0 to 100, with higher scores indicating greater perceived distress. Hopelessness was assessed through self-assessment by asking callers to rate their sense of hopefulness about the future, with higher scores indicating lower levels of hopelessness (21). In this study, following the conventional classification criteria used in previous hotline research, distress and hopelessness scores were dichotomized based on the median values (22): distress was categorized into a high group (80–100) and a low group (0–79); hopelessness was categorized into a high group (0–29) and a low group (30–100).

The hotline collected demographic characteristics during initial assessments, including gender, age, marital status (unmarried, married, divorced, widowed), education duration, history of psychiatric diagnoses (yes/no), and current treatment status (yes/no).

### 2.3 Statistical analysis

Statistical analyses were performed using SPSS 26.0. Categorical variables (age, education duration, marital status, call time, psychiatric history, and treatment status) were summarized as frequencies (n) and percentages (%). *χ*² tests compared differences in characteristics across age and gender groups. The presence or absence of suicidal ideation, hopelessness, psychological distress, and depression severity serving as dependent variables. Multivariate logistic regression was conducted to identify mental health correlates, incorporating demographics, psychiatric history, and treatment status as predictors. Statistical significance was set at *P*<0.05.

## 3 Results

Among 9,002 unemployed callers, 5,426 were female (60.3%) and 3,576 were male (39.7%); calls were most frequent in the morning (8:00-12:00, 33.7%), followed by the afternoon (13:00-18:00, 28.6%), early morning (0:00-7:00, 25.8%), and evening (19:00-23:00, 11.9%). The most frequently reported issues among unemployed callers were psychiatric concerns (28.5%), followed by family relationship problems (27.0%), depression (25.5%) and interpersonal problems outside the family (24.0%). Among callers aged 19-29, the proportions of males (*χ*²=45.37*, P*<0.001), those who were unmarried (*χ*²=2320.19*, P*<0.001), those without a prior mental health diagnosis (*χ*²=23.77*, P* < 0.001), and those who had never sought help before (*χ*²= 14.69*, P*<0.001) were significantly higher than those among callers aged 30-59. Among callers aged 30-39, the proportion of those with 16 or more years of education (*χ*²=8.62*, P*<0.01) was significantly higher than among those aged 19–29 and 40-59.

Among callers aged 40-59, the proportions of females (*χ*²=45.37*, P*<0.001), those who were married, divorced, or widowed (*χ*²=2320.19*, P* < 0.001), individuals with i15 years of education (*χ*²=8.62*, P*<0.01), those with a prior psychiatric diagnosis (*χ*²=23.77*, P*<0.001), and those who had received treatment (*χ*²=14.69*, P*<0.001) were significantly higher than among those aged 19-39. Callers aged 19–29 were more likely to call during 19:00-23:00, those aged 30–39 were more likely to call during 13:00-18:00, and those aged 40–59 predominantly called during 0:00-12:00 (*χ*²=16.58*, P*<0.01). Callers aged 19–29 were significantly more likely than those aged 30–59 to report issues related to non-family interpersonal relationships (*χ*²=8.03*, P*<0.01), depression (*χ*²=7.48*, P*<0.01), and psychiatric symptoms (*χ*²=7.04*, P*<0.01), as shown in **Table 1**.

**Table 1:** Comparison of general characteristics of unemployed hotline callers by age group (n (%)).

Variable	Total (n=9002)	19–29 years (n=6333)	30-39years (n=2222)	40-59years (n=447)	*X* ^2^	*P*
Gender					45.37	0.000
Female	5426 (60.3)	3675 (58.0)	1452 (65.3)	299 (66.9)		
Male	3576 (39.7)	2658 (42.0)	770 (34.7)	148 (33.1)		
Marital status					2320.19	0.000
Unmarried	6570 (73.2)	5517 (87.3)	961 (43.4)	92 (20.6)		
Married	1780 (19.8)	644 (10.2)	903 (40.7)	233 (52.2)		
Divorced/Widowed	631 (7.0)	158 (2.5)	352 (15.9)	121 (27.1)		
Years of education					8.62	0.013
≤15years	5794 (64.8)	4077 (64.8)	1404 (63.8)	313 (71.1)		
≥16years	3141 (35.2)	2219 (35.2)	795 (36.2)	127 (28.9)		
History of psychiatric diagnoses					23.77	0.000
Yes	4567 (50.9)	3110 (49.2)	1204 (54.3)	253 (57.0)		
No	4409 (49.1)	3205 (50.8)	1013 (45.7)	191 (43.0)		
Current treatment					14.69	0.001
Yes	7651 (85.5)	5421 (86.1)	1879 (84.9)	351 (79.6)		
No	1301 (14.5)	877 (13.9)	334 (15.1)	90 (20.4)		
Time of call					16.58	0.011
00:00-07:00	2866 (25.8)	1770 (25.2)	764 (26.2)	332 (29.0)		
08:00-12:00	3737 (33.7)	2380 (33.9)	960 (32.9)	397 (34.7)		
13:00-18:00	3167 (28.6)	2003 (28.5)	860 (29.5)	304 (26.6)		
19:00-23:00	1320 (11.9)	876 (12.5)	334 (11.4)	110 (9.6)		
Presenting problem						
Family relationship issues	2435 (27.0)	1739 (27.5)	578 (26.0)	118 (26.4)	1.85	0.397
Interpersonal issues outside family	2158 (24.0)	1570 (24.8)	486 (21.9)	102 (22.8)	8.03	0.018
Work-related issues	749 (8.3)	529 (8.4)	186 (8.4)	34 (7.6)	0.32	0.854
Economic issues	772 (8.6)	542 (8.6)	197 (25.5)	33 (4.3)	1.05	0.591
Academic issues	246 (2.7)	186 (2.9)	49 (2.2)	11 (2.5)	3.45	0.178
Depression	2294 (25.5)	1664 (26.3)	519 (23.4)	111 (24.8)	7.48	0.024
Psychiatric symptoms	2562 (28.5)	1840 (29.1)	617 (27.8)	105 (23.5)	7.04	0.030
Other negative events	685 (7.6)	488 (7.7)	153 (6.9)	44 (9.8)	4.91	0.086

Data missing in some variables. Because of missing data, for most variables, Percentages do not total 100%.

Among female callers, the proportions of those aged 30-59 (*χ*²=45.37*, P*<0.001), those who were married, divorced, or widowed (*χ*²=407.809*, P* < 0.001), those who had 16 or more years of education (*χ*²=259.76*, P*<0.001), and those reporting family relationship issues (*χ*²=153.79*, P*<0.001), non-family interpersonal problems (*χ*²=144.82*, P*<0.001), work-related concerns (*χ*²=21.134*, P*<0.001), economic difficulties (*χ*²=19.916*, P*<0.001), academic issues (*χ*²=103.98*, P*<0.001), depression (*χ*²=150.12*, P*<0.001), psychiatric symptoms (*χ*²=11.09*, P* < 0.001), and other negative life events (*χ*²=9.950*, P*<0.001) were significantly higher than those among males. The proportions of males aged 19-29 (*χ*²=45.37*, P*<0.001), those who were unmarried (*χ*²=407.809*, P*<0.001), and those with ≤15 years of education (*χ*²=259.76*, P*<0.001) were significantly higher than among female callers, as shown in **Table 2**.

**Table 2:** Comparison of general characteristics of unemployed hotline callers by gender (n (%)).

Variable	Female (n=5426)	Male (n=3576)	*χ* ^2^	*P*
Age group			45.376	0.000
19–29 years	3675 (67.7)	2658 (74.3)		
30–39 years	1452 (26.8)	770 (21.5)		
40–59 years	299 (5.5)	148 (4.1)		
Marital status			407.809	0.000
Unmarried	3568 (65.9)	3002 (84.2)		
Married	1432 (26.4)	348 (9.8)		
Divorced/Widowed	414 (7.6)	217 (6.1)		
Years of education			257.87	<0.001
≤15years	3132 (58.2)	2662 (74.8)		
≥16years	2245 (41.8)	896 (25.2)		
History of psychiatric diagnoses			34.63	<0.001
Yes	2890 (53.4)	1677 (47.1)		
No	2522 (46.6)	1887 (52.9)		
Current treatment			1.347	0.246
Yes	4592 (85.1)	3059 (86.0)		
No	803 (14.9)	498 (14.0)		
Time of call			0.384	0.944
00:00-07:00	1570 (25.6)	1086 (25.4)		
08:00-12:00	2072 (33.8)	1433 (33.5)		
13:00-18:00	1742 (28.5)	1239 (29.2)		
19:00-23:00	739 (12.1)	514 (12.0)		
Presenting problem				
Family relationship issues	2233 (41.2)	1013 (28.3)	153.790	<0.001
Interpersonal issues outside family	2051 (37.8)	916 (25.6)	144.817	<0.001
Work-related issues	726 (13.4)	363 (10.2)	21.134	<0.001
Economic issues	712 (13.1)	358 (10.0)	19.916	<0.001
Academic issues	2135 (39.3)	1032 (28.9)	103.982	<0.001
Depression	2361 (43.5)	1097 (30.7)	150.116	<0.001
Psychiatric symptoms	602 (11.1)	319 (8.9)	11.094	<0.001
Other negative events	229 (4.2)	105 (2.9)	9.950	<0.001

Because of missing data, for most variables, Percentages do not total 100%.

Based on the median scores of hopelessness (29), psychological distress (80), and depression (50), these variables were dichotomized and used as dependent variables. Demographic variables (gender, age, years of education, marital status), previous psychiatric diagnoses, and current treatment status were included as independent variables in multivariate logistic regression. The results showed that unemployed female callers were more likely to experience suicidal ideation (OR=1.50, 95% CI:1.28-1.75). Callers aged 30-39 (OR=0.67,95% CI: 0.56-0.80), 40-59 (OR=0.45,95% CI:0.34-0.61), and those with a history of psychiatric diagnosis (OR=0.66, 95%CI:0.57-0.78) were less likely to report suicidal ideation. Higher levels of hopelessness were observed among female callers (OR=1.13, 95% CI:1.04-1.24), those aged 30-39 (OR=1.13, 95% CI:1.01-1.26), unmarried individuals (OR=1.30, 95% CI:1.16-1.47), divorced individuals (OR=1.63, 95% CI:1.35-1.97), and those with a prior psychiatric diagnosis (OR = 1.18, 95% CI: 1.08-1.30). Callers who were divorced (OR=1.39, 95% CI:1.16-1.66) or had a previous psychiatric diagnosis (OR=1.11,95% CI:1.01-1.22) reported higher levels of psychological distress. Higher levels of depression were found among female callers (OR=1.09, 95% CI:1.00-1.19), those who were divorced (OR=1.57, 95% CI:1.31-1.87), and those with a prior psychiatric diagnosis (OR=1.11, 95% CI: 1.02-1.21). Lower levels of hopelessness were reported by callers with 16 or more years of education (OR=0.68, 95% CI: 0.64-0.76), and those who called between 8:00-12:00 (OR = 0.68, 95% CI: 0.61-0.76), 13:00-18:00 (OR=0.71, 95% CI: 0.63-0.80), or 19:00-23:00 (OR=0.80, 95% CI: 0.69-0.93). Lower psychological distress was also associated with having 16 or more years of education (OR=0.89, 95% CI: 0.81-0.97) and calling between 13:00-18:00 (OR=0.71, 95% CI: 0.63-0.80). Callers aged 30-39 (OR=0.88, 95% CI: 0.79-0.98), aged 40-59 (OR=0.45, 95% CI: 0.36-0.56), and those with 16 or more years of education (OR=0.73, 95% CI: 0.67-0.79) exhibited lower levels of depression, as shown in **Table 3**.

**Table 3:** Multivariate logistic regression results of factors affecting mental health among unemployed hotline callers.

Factor	Suicidal ideation (=1)	High hopelessness (=1)	High psychological distress (=1)	High depression severity (=1)
Gender (Female=1)	1.50 (1.28~1.75)***	1.13 (1.04~1.24)*	0.94 (0.86~1.03)	1.09 (1.00~1.19)*
Age				
30-39 (=1)	0.67 (0.56~0.80)***	1.13 (1.01~1.26)*	0.98 (0.88~1.09)	0.88 (0.79~0.98)*
40-59 (=2)	0.45 (0.34~0.61)***	0.98 (0.80~1.21)	0.88 (0.72~1.08)	0.45 (0.36~0.56)***
Education (≥16 years=1)	1.07 (0.92~1.24)	0.68 (0.64~0.76)***	0.89 (0.81~0.97)*	0.73 (0.67~0.79)***
Marital status				
Unmarried (=1)	0.92 (0.75~1.12)	1.30 (1.16~1.47)***	0.93 (0.82~1.04)	1.06 (0.94~1.20)
Divorced/Widowed (=2)	0.96 (0.73~1.28)	1.63 (1.35~1.97)***	1.39 (1.16~1.66)***	1.57 (1.31~1.87)***
History of psychiatric diagnoses (=1)	0.66 (0.57~0.78)***	1.18 (1.08~1.30)***	1.11 (1.01~1.22)*	1.11 (1.02~1.21)*
Currently Receiving Treatment (=1)	0.84 (0.69~1.02)	0.91 (0.80~1.04)	0.96 (0.85~1.09)	0.95 (0.84~1.08)
Time of call				
08:00-12:00 (=1)	1.01 (0.84~1.22)	0.68 (0.61~0.76)***	0.91 (0.81~1.01)	1.03 (0.93~1.15)
13:00-18:00 (=2)	1.04 (0.86~1.27)	0.71 (0.63~0.80)***	0.86 (0.77~0.96)*	0.97 (0.86~1.08)
19:00-23:00 (=3)	0.89 (0.70~1.14)	0.80 (0.69~0.93)*	0.90 (0.79~1.04)	0.89 (0.78~1.03)

*P<0.05, **P<0.01, ***P<0.001.

## 4 Discussion

This study found that the majority of unemployed callers to the psychological support hotline were women, with their primary concerns focusing on depression, family relationships, and interpersonal issues. Regression analysis revealed that unemployed female callers were more likely to exhibit suicidal ideation and experience higher levels of hopelessness and depression.

Results are consistent with previous studies: women are more likely to express emotions and seek psychological support through hotlines (23–25), and this tendency was shaped by gender role socialization in which women are encouraged to share emotions, build relationships, and seek help, whereas men are often socialized to solve problems independently and suppress emotional expression (26). Previous research indicates that unemployed men face a higher risk of suicide (27), while unemployed women are more prone to emotional disorders (28), psychological distress (29), and poorer overall mental health (30), which contrasts with the higher suicidal ideation observed among unemployed women in this study. This discrepancy may partly reflect gender differences in help-seeking behavior and modes of crisis expression. Within the East Asian collectivist culture, the concept of “face” and the prevailing culture of shame lead men to suppress negative emotions such as feelings of failure and vulnerability, consequently reducing their willingness to seek psychological help (31, 32). In contrast, women are more likely to adopt emotionally expressive coping strategies (33, 34), making their psychological distress more readily identifiable and addressable in hotline settings. In terms of crisis expression, women are more inclined toward “expressive crises,” characterized by verbal disclosure of emotional pain and suicidal ideation, whereas men tend to exhibit “behavioral crises,” manifesting in impulsive and potentially lethal actions under emotional suppression (35). This may result in an underestimation of men’s true psychological crises during hotline interventions. Furthermore, employment serves as a significant protective factor for women’s mental health (36). Balancing multiple social roles helps individuals accumulate psychological resources (37), such as economic independence, expanded social support networks, enhanced self-esteem, and self-efficacy. Unemployment disrupts this balance, placing a dual burden on women of lost income and traditional caregiving responsibilities (38), thereby increasing psychological stress. In collectivist cultures, female unemployment may be perceived as a failure in role fulfillment, triggering feelings of inadequacy and guilt, and elevating the risk of depression (37). Disruption of occupational identity may also weaken women’s social networks and access to external support, thereby compromising their psychological resilience.

The results also showed that unemployed individuals aged 19–29 were significantly overrepresented in psychological hotline calls compared to other age groups, with their primary concerns focused on interpersonal difficulties, depression, and psychiatric symptoms. Regression analysis revealed that, compared with unemployed individuals aged 19-29, those aged 30–59 were less likely to report suicidal ideation and exhibited lower levels of depressive symptoms. According to Erikson’s eight-stage theory of psychosocial development, individuals in this age range are transitioning from “identity versus role confusion” to “intimacy versus isolation,” during which self-identity is still forming and social roles remain unstable (39). Career uncertainty during this phase may destabilize their sense of identity and expectations for the future, triggering psychological distress such as anxiety and depression (40). Young adults often lack mature emotion regulation strategies and robust social support systems, making them more vulnerable to emotional distress and psychological crises in the face of unemployment. Previous studies have shown that financial insecurity is strongly associated with psychological stress (41), and young individuals may internalize unemployment as a marker of social failure and self-rejection (42), leading to diminished self-esteem and social withdrawal. Recent studies based on linguistic analysis of Weibo posts have demonstrated that unemployed graduates exhibit a higher frequency of negative emotion words on social media, reflecting pronounced psychological distress and feelings of loneliness (43). Notably, in this study, the interpersonal concerns voiced by young callers predominantly involved relationships outside the family, contrasting with prior research that highlighted conflicts with family members (44). This discrepancy may be explained by the youth’s greater focus on peer interactions in their pursuit of employment and social validation, which may lead to underreporting of family-related issues during hotline calls. The psychological hotline, characterized by its anonymity and low threshold for access, serves as a vital support channel for this population.

Furthermore, the current study found that unmarried individuals accounted for a higher proportion of unemployment-related calls to the psychological support hotline compared to married individuals. Moreover, unemployed callers who were unmarried, divorced, or widowed reported significantly higher levels of hopelessness, distress, and depressive symptoms than married callers. Cross-cultural studies have shown that unmarried individuals are more susceptible to psychological symptoms such as depression and anxiety compared to those who are married (45). Stable intimate relationships, particularly spousal partnerships, serve as critical resources for coping with stress by enhancing emotional regulation, buffering the impact of stressors such as unemployment (46), providing emotional support, and contributing to financial stability (47). Married individuals may derive emotional and functional support through their family roles, partially compensating for the loss of occupational identity. In contrast, unemployed unmarried individuals may lack stable support networks (48), making them more vulnerable to loneliness and social alienation (49), thereby intensifying psychological fragility. Those who are financially dependent on their parents are particularly prone to experiences of stigma, further increasing their risk for mental health problems. As a result, they are more likely to seek psychological support through hotlines as a form of substitute support or emotional expression (50).

Regression analysis indicated that unemployed callers with 16 or more years of education reported lower levels of hopelessness, psychological distress, and depressive symptoms. Hence, educational attainment is a key determinant of mental health, with higher levels of education being associated with greater psychological literacy and a reduced risk and severity of mental health problems (51, 52). OECD data shows that for every year of education lost, the risk of developing depression after unemployment increases by 7% (53). Higher education fosters a problem-solving mindset toward health, encourages proactive use of medical and preventive services (54), and strengthens individuals’ capacity to recognize psychological distress and manage stress effectively (55). Moreover, education enhances self-efficacy and facilitates the development of social support networks, thereby improving resilience and hopefulness in the face of unemployment, and mitigating negative emotional responses (56).

Regression analysis also revealed that callers with a history of psychiatric diagnoses exhibited lower rates of suicidal ideation, but reported higher levels of hopelessness, psychological distress, and depression. Individuals with a history of psychiatric diagnoses face an unemployment rate 1.8 times higher than the general population (7) and experience a 40% longer reemployment period. Unemployment not only exacerbates preexisting symptoms of anxiety and depression but also undermines compensatory mechanisms such as social support systems, leading to a collapse in self-regulation. Financial strain and identity crises further diminish individuals’ sense of hope (36). The lower incidence of suicidal ideation among these individuals may be attributed to their integration into the mental healthcare system, where they receive professional interventions such as pharmacotherapy, psychotherapy, and crisis management, which have been proven to effectively reduce suicide risk (57, 58). Furthermore, the disease management process often provides access to relatively stable support systems, including family support, regular follow-up care, and community resources, which contribute to a reduced risk of suicide among callers (59, 60). However, hopelessness, psychological distress, and the severity of depression are themselves significant risk factors for suicide. Even if current suicidal ideation is low, continuous monitoring and reinforced interventions remain necessary to prevent risk accumulation and potential crisis escalation.

Regression analysis further showed that callers who contacted the hotline between 8:00 and 23:00 exhibited significantly lower levels of hopelessness compared to those calling between 0:00 and 7:00. Individuals are more likely to contact mental health hotlines when alone (51), and nighttime is associated with heightened subjective loneliness and negative emotions; the reduced accessibility of social support during these hours may intensify feelings of hopelessness (61). During the day, unemployed individuals have greater access to online skills training and community activities, which facilitate social interaction and external support, thereby alleviating negative emotions (62). Moreover, research indicates that individuals with stronger coping abilities and proactive tendencies are more likely to seek help during daytime hours (63), which may further influence the overall mental state of callers during this period.

This study has several limitations. Firstly, the data were obtained from a psychological support hotline, which may introduce selection bias and reporting bias, as callers are all self-initiated help-seekers, and underreporting may occur particularly regarding stigmatized or sensitive issues. Secondly, the findings are based on cross-sectional data, limiting the ability to infer causal relationships. Future research should adopt longitudinal designs to further explore the dynamic relationship between unemployment and mental health. Additionally, the study period encompassed both the COVID-19 pandemic and the post-pandemic phases. While the pandemic likely impacted individuals’ psychological states and help-seeking behaviors, this study did not conduct an in-depth analysis of its effects across these phases. Future studies could further investigate the influence of the pandemic and related factors on mental health at various phases. Finally, socioeconomic variables such as duration of unemployment, specific occupational categories, and economic status were not included in this study. Subsequent research could incorporate these factors to provide more comprehensive mental health intervention strategies.

## 5 Conclusions

The current study, based on an analysis of unemployed callers to the Beijing Psychological Support Hotline, found that women, young adults aged 19-29, and individuals who are unmarried or divorced demonstrate elevated psychological distress or suicide risk. Among them, callers with a history of psychiatric diagnoses showed lower levels of suicidal ideation but higher levels of hopelessness and depression. Hotline operators should receive regular specialized training to enhance their sensitivity and capacity for identifying and intervening with high-risk individuals. Emphasizing the heterogeneity of psychological distress among callers can improve the precision and professionalism of service responses, optimize resource allocation and support mechanisms, and ultimately alleviate psychological burdens and promote mental health recovery.

## Data Availability

The original contributions presented in the study are included in the article/supplementary material. Further inquiries can be directed to the corresponding author.
