# Correction: Analysis of characteristics among unemployed callers to the psychological support hotline in Beijing

**DOI:** 10.3389/fpsyt.2025.1706852

**Published:** 2025-09-30

**Authors:** Xingxue Li, Zikang Liu, Tong Wu, Zhe Yuan, Wei Wang, Liting Zhao, Shuchang Yang, Hong Liang

**Affiliations:** ^1^ Beijing Suicide Research and Prevention Center, Beijing Huilongguan Hospital, Beijing, China; ^2^ World Health Organization Collaborating Center for Research and Training in Suicide Prevention, Beijing, China; ^3^ Graduate School, Chengde Medical University, Chengde, China; ^4^ Faculty of Psychology, Tianjin Normal University, Tianjin, China; ^5^ Mental Health Education Center, Baoding Institute of Technology, Baoding, Hebei, China; ^6^ Psychology and Language Science, Univeristy College London, London, United Kingdom

**Keywords:** psychological support hotline, unemployment, mental health, characteristics, risk factors

An incorrect **Funding** statement was provided. This originally read: “The author(s) declare that no financial support was received for the research and/or publication of this article.” However, this research was funded by “Beijing Natural Science Foundation, China”. The correct Funding statement reads:

“This work was supported by Beijing Natural Science Foundation, China, under Grant No. 7182074. The project title is “Abnormal Serum Levels of NCAM/PSA-NCAM as Markers for Cognitive Impairment in Schizophrenia and Their Imaging Evidence”.”

The original version of this article has been updated.

